# Draft Genome Sequence of Strain Q-1, an Iodide-Oxidizing Alphaproteobacterium Isolated from Natural Gas Brine Water

**DOI:** 10.1128/genomeA.00659-14

**Published:** 2014-07-03

**Authors:** Ayaka Ehara, Haruo Suzuki, Yu Kanesaki, Hirofumi Yoshikawa, Seigo Amachi

**Affiliations:** a Graduate School of Horticulture, Chiba University, Matsudo City, Chiba, Japan; b Graduate School of Science and Engineering, Yamaguchi University, Yamaguchi, Japan; c Genome Research Center, NODAI Research Institute, Tokyo University of Agriculture, Setagaya-ku, Tokyo, Japan; d Department of Bioscience, Tokyo University of Agriculture, Setagaya-ku, Tokyo, Japan

## Abstract

Here we report the draft genome sequence of strain Q-1, an iodide (I^−^)-oxidizing heterotrophic bacterium in the class *Alphaproteobacteria* isolated from natural gas brine water. The genome sequence contained a multicopper oxidase gene probably responsible for iodide oxidation. A photosynthetic gene cluster was found but genes for carbon-fixation were absent.

## GENOME ANNOUNCEMENT

Iodide-oxidizing bacteria are able to oxidize iodide (I^−^) to molecular iodine (I_2_) and have been isolated from natural gas brine water containing very high concentrations (60 µM to 1.2 mM) of iodide (1). They are aerobic heterotrophic bacteria in the class *Alphaproteobacteria* and can also be enriched from natural seawater supplemented with iodide, due to much higher I_2_ tolerance than the other heterotrophic bacteria in seawater (2). Recently, such bacteria have been found to be involved in microbial clogging of well pipes in iodine-producing facilities (3, 4). Strain Q-1 was isolated from brine water in Miyazaki, Japan, and was phylogenetically related to the halophilic photosynthetic bacterium *Rhodothalassium salexigens*, with a 16S rRNA gene sequence similarity of 90% (1). This strain showed a strong iodide-oxidizing enzyme activity, which was later found to be one of multicopper oxidases (5). Here we report the draft genome sequence of strain Q-1.

Strain Q-1 was grown in Marine Broth 2216 (Becton Dickinson, Sparks, MD) and DNA was extracted using a DNeasy Blood & Tissue kit (Qiagen, Hilden, Germany). A DNA library with a median insert size of 200 bp was constructed. The library was sequenced on a genome analyzer II (Illumina, San Diego, CA), with a read length of 75 bp, trimmed to 40 bp prior to assembly. This produced a total of 11,698,620 paired-end read sequences with 468 Mb of total read data. Genome assembly was performed using SPAdes version 3.0.0 (6), yielding a collection of 109 contigs at least 200 bp long, with an *N*_50_ size of 102,443 bp. This assembly had 3,085,726 bp, with a genome coverage of 152× and a G+C content of 56.1%. Genome annotation was performed using Prokka 1.8 (7), yielding a total of 2,788 protein-coding genes (CDSs), 46 tRNA genes, and one copy of the 16S-23S-5S rRNA gene.

The genome contains one multicopper oxidase gene (*ioxA*), which was previously found to be involved in iodide oxidation by this strain (5). The genome also contains one continuous 45,135-bp-long photosynthetic gene cluster. The organization of the genes in the cluster is *pufCMLAB*-*bchZYXC*-*crtFEDC*-open reading frame (ORF)-*crtBI*-ORF-*bchODI*-ORFs-*bchP*-*pucC*-*bchG*-*ppsR*-*ppaA*-*bchFNBHLM*-*lhaA*-*puhABC*-ORF*-acsF-puhE-hemA-tspO*. There are no autotrophic CO_2_ fixation pathway genes such as those for ribulose-1,5-bisphosphate carboxylase/oxygenase and components of the reductive tricarboxylic acid (TCA) cycle in the draft genome sequence. A key gene encoding phosphofructokinase for glycolysis is absent, but a complete set of genes for the TCA cycle, pentose phosphate pathway, and Entner-Doudoroff pathway are predicted. The genome contains nitrate reductase genes (*nar*) but not nitrite reductase (*nir*) and nitrogenase (*nif*) genes. A complete set of genes for the type II secretion system (*gsp*) as well as for the flagellar system (*fli, flg*, and *mot*) was identified. Various proteins that may be involved in aerobic metabolism were identified, including NADH dehydrogenase, succinate dehydrogenase, cytochrome-*c* oxidase, catalase-peroxidase, and superoxide dismutase. These results suggest that strain Q-1 is one of the aerobic anoxygenic phototrophic bacteria.

### Nucleotide sequence accession numbers.

This whole-genome shotgun project has been deposited at DDBJ/EMBL/GenBank under the accession no. BAYV00000000. The version described in this paper is the first version, BAYV01000000.
